# Determinants of prognosis after reperfusion therapy in acute ischemic stroke and development of a predictive model

**DOI:** 10.3389/fneur.2026.1756705

**Published:** 2026-07-30

**Authors:** Chaoqing Yu, Peng Wang, Zedong Cheng, Wandi Chen, Rongbo Qu

**Affiliations:** 1 The Second Clinical Medical College, Binzhou Medical University, Yantai, Shandong, China; 2 Neurointerventional Department, Yantai Affiliated Hospital of Binzhou Medical University, Yantai, Shandong, China

**Keywords:** acute ischemic stroke, functional outcome, nomogram, prognostic model, reperfusion therapy, ΔmRS

## Abstract

**Introduction:**

Acute ischemic stroke (AIS) remains a leading cause of death and disability worldwide. Despite improvements in recanalization rates with intravenous thrombolysis and mechanical thrombectomy, a substantial proportion of patients experience poor functional recovery. Conventional endpoints based solely on the 3-month modified Rankin Scale (mRS) may be affected by heterogeneity in baseline functional status. This study used ΔmRS as a directional indicator of functional improvement and developed an admission-based pre-reperfusion model to predict poor functional recovery after reperfusion therapy.

**Methods:**

This single-center retrospective cohort study included 374 patients with AIS who underwent reperfusion therapy at Yantai Affiliated Hospital of Binzhou Medical University between October 2023 and December 2024. ΔmRS was calculated as the admission mRS score minus the 3-month mRS score. Patients were classified as having significant functional improvement when ΔmRS was ≥2 and as having non-significant improvement or poor functional recovery when ΔmRS was <2. Patients were randomly divided into a training cohort and a validation cohort at a ratio of 7:3. Predictors were selected using LASSO regression and multivariable logistic regression, and a nomogram was subsequently developed.

**Results:**

Poor functional recovery occurred in 124 of 374 patients (33%). Higher admission National Institutes of Health Stroke Scale score, lower baseline Alberta Stroke Program Early CT Score, elevated admission blood glucose level, higher age-adjusted Charlson Comorbidity Index score, and concomitant atrial fibrillation were independently associated with poor functional recovery. The model achieved an area under the receiver operating characteristic curve of 0.832 in the training cohort and 0.799 in the validation cohort, with acceptable calibration and potential net clinical benefit.

**Discussion:**

The ΔmRS-based nomogram may provide a preliminary and clinically interpretable tool for early risk stratification in patients with AIS undergoing reperfusion therapy. ΔmRS should be regarded as a complementary rather than replacement outcome measure, and the model requires further validation in larger, multicenter, and external cohorts.

## 1 Introduction

Acute ischemic stroke (AIS) remains one of the leading causes of death and long-term disability worldwide, imposing a substantial health and socioeconomic burden on both individuals and healthcare systems (1, 2). With population aging and the increasing prevalence of vascular risk factors, the incidence of AIS continues to rise in many countries (2, 3). Rapid restoration of cerebral blood flow is the cornerstone of modern AIS management, and reperfusion therapies, including intravenous thrombolysis (IVT) and mechanical thrombectomy (MT), have substantially improved outcomes in selected patients by salvaging ischemic but potentially viable brain tissue (4–6).

Despite major advances in reperfusion therapy and increasing rates of successful recanalization, clinical outcomes after AIS remain highly heterogeneous. In routine practice, a considerable proportion of patients still fail to achieve meaningful neurological recovery even after technically successful reperfusion (7, 8). This phenomenon suggests that restoration of vessel patency alone is insufficient to determine prognosis, and that post-reperfusion recovery is influenced by multiple interacting factors, including baseline neurological deficit, ischemic core burden, metabolic status, comorbidity burden, collateral circulation, and treatment-related factors (9, 10). Therefore, identifying key determinants of functional recovery and developing clinically applicable prognostic models remain important goals in AIS research.

At present, most prognostic studies and prediction models for AIS after reperfusion therapy are based on conventional outcome definitions, such as 3-month functional independence measured by a modified Rankin Scale (mRS) score ≤2, poor functional outcome, mortality, or treatment-related complications (11–13). These models, often constructed using variables such as age, baseline National Institutes of Health Stroke Scale (NIHSS) score, Alberta Stroke Program Early CT Score (ASPECTS), blood glucose level, and comorbidities, have provided useful tools for clinical risk stratification (12, 13). However, such measures primarily describe the patient’s final functional status at a fixed time point and provide only limited information regarding the magnitude of functional improvement or the trajectory of recovery after treatment. This issue is particularly relevant in patients undergoing reperfusion therapy, in whom baseline neurological severity, pre-stroke functional reserve, and tissue salvageability may vary substantially. As a result, the same 90-day mRS score may reflect very different recovery processes and treatment gains across individuals. Therefore, reliance on a fixed endpoint alone may not fully capture individualized recovery potential or the dynamic nature of post-treatment functional change (9, 14). Against this background, outcome measures based on functional change before and after treatment may offer a complementary perspective for stroke recovery research. In this study, ΔmRS was calculated as admission mRS minus 3-month mRS. Thus, ΔmRS was used as a directional indicator of functional improvement rather than a non-directional measure of change. This definition allowed us to distinguish functional improvement from no improvement or deterioration during the 3-month follow-up. Unlike the conventional 90-day mRS, which primarily reflects the final functional state at a single time point, ΔmRS focuses on the magnitude and direction of functional improvement from admission to the 3-month follow-up. This characteristic may provide additional information in populations with heterogeneous baseline neurological severity (10, 14). Importantly, ΔmRS is not intended to replace established outcome measures such as the traditional 90-day mRS; rather, it is introduced here as a complementary measure that may help characterize individualized recovery trajectories and potential treatment benefit from a dynamic perspective. Although studies specifically using ΔmRS in prognostic assessment after stroke remain limited, the broader emphasis in stroke recovery research on functional change and recovery processes provides a reasonable conceptual basis for its application in the present study.

Meanwhile, although predictive modeling in AIS has progressed rapidly, currently available models still face several practical limitations. Some models rely on complex structures or variables that are not always readily available in emergency settings, while others do not sufficiently account for differences in baseline function or the extent of post-treatment improvement (12, 13). Therefore, there remains a need for a simple, interpretable, and clinically applicable model that can help physicians estimate the likelihood of poor functional recovery after reperfusion therapy using routinely accessible data.

Therefore, we retrospectively analyzed routinely available admission clinical, laboratory, and imaging data from AIS patients who underwent reperfusion therapy. The aim was to identify early predictors of poor functional recovery and to develop an admission-based pre-reperfusion prediction model. The model was designed for early risk stratification before or at the initiation of reperfusion therapy, rather than for prediction after procedural outcomes were known. Compared with conventional endpoint-based models, our approach emphasizes the magnitude of neurological change before and after treatment and may therefore better reflect individualized recovery potential. In addition, the proposed model was developed using routinely available variables and visualized as a nomogram, which may enhance its practicality and interpretability in real-world clinical settings. Prognostic models for AIS reperfusion therapy using ΔmRS as the primary indicator of functional improvement remain limited, and the present study may provide a useful complement to conventional endpoint-based prognostic frameworks. The performance of the model was evaluated in terms of discrimination, calibration, and potential clinical utility, with the aim of providing a practical tool for early risk stratification and personalized management after reperfusion therapy.

## 2 Materials and methods

### 2.1 General data

**Figure 1:**
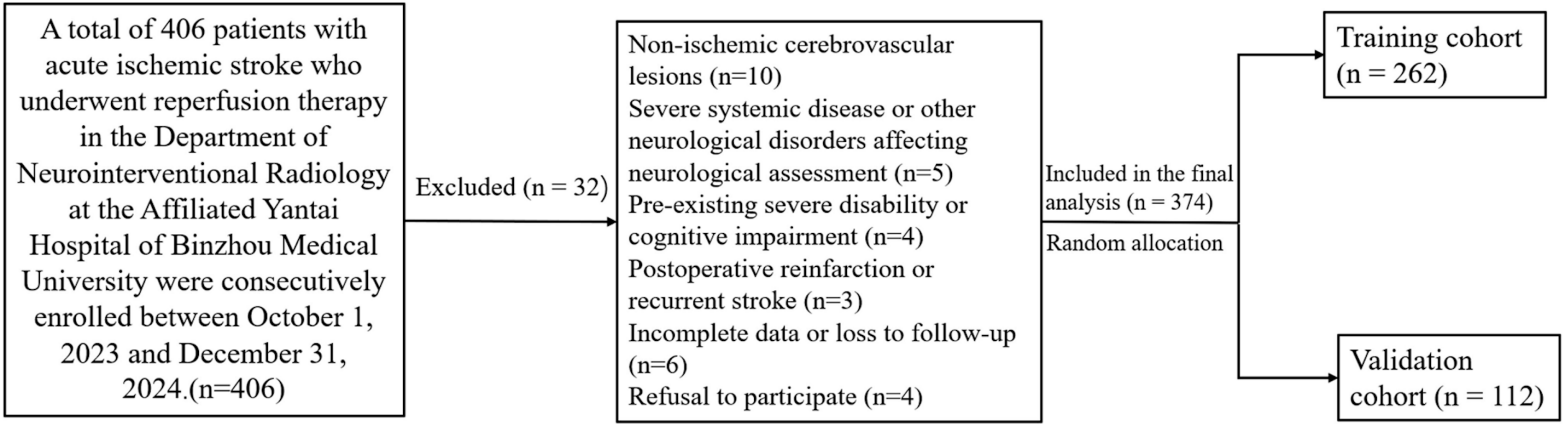
Flowchart of patient selection.

This was a single-center retrospective cohort study. A total of 406 consecutive patients with AIS who underwent reperfusion therapy at the Department of Neurointerventional Radiology, Yantai Affiliated Hospital of Binzhou Medical University, between October 1, 2023 and December 31, 2024, were initially screened. To improve cohort homogeneity and reduce the risk of selection bias, predefined inclusion and exclusion criteria were established based on relevant literature and current clinical guidelines. After systematic screening, 374 patients were finally included in the analysis. The patient selection process is shown in Figure 1.

#### 2.1.1 Inclusion criteria

1. Diagnostic Criteria: Imaging-confirmed AIS excluding intracranial hemorrhagic lesions; imaging evidence of acute occlusion of the responsible vessel was present,responsible vessels being the internal carotid artery, M1/M2 segment of the middle cerebral artery or basilar artery, or vertebral artery.
2. Time-to-Treatment Requirement: Time from symptom onset to initiation of reperfusion therapy must not exceed 24 h. For patients undergoing delayed thrombectomy, imaging criteria from the DAWN or DEFUSE-3 trials must be met, indicating a salvageable penumbra characterized by a small infarct core and large perfusion defect, along with clinical-imaging mismatch (15).
3. Baseline functional status: Pre-stroke functional status was relatively preserved, defined as a pre-stroke mRS score ≤2, to minimize bias arising from pre-existing disability in outcome assessment; The pre-stroke mRS score was determined based on the patient’s functional status within the 2 weeks prior to stroke onset, as documented in the medical records or obtained from patient or family interviews. This criterion was used to reduce the potential influence of baseline functional heterogeneity on the assessment of post-treatment recovery. The pre-stroke mRS score was used only to assess pre-existing disability for eligibility and was not used in the calculation of ΔmRS.
4. Data Completeness: Patients must have complete clinical records, imaging data, and follow-up information, with mRS scores obtainable at admission and at 3 months.
5. Informed Consent: Patients or their immediate family members must sign an informed consent form agreeing to undergo revascularization therapy and subsequent clinical follow-up.

#### 2.1.2 Exclusion criteria

1. Imaging evidence of non-ischemic cerebrovascular events, including intracerebral hemorrhage, subarachnoid hemorrhage, or mixed stroke.
2. Presence of other conditions affecting neurological assessment or prognosis determination, including non-vascular brain lesions such as brain tumors or demyelinating diseases, severe systemic diseases including end-stage liver or renal failure, advanced malignancy, severe infection with organ dysfunction, or hemodynamic shock, as well as concomitant neurological disorders such as Parkinson’s disease or epilepsy, all of which could substantially affect neurological evaluation or short-term prognosis.
3. History of severe disability or clinically documented cognitive impairment: Patients with a pre-stroke mRS score >2, those bedridden long-term for other reasons, or those with dementia or severe cognitive dysfunction documented in the medical records and considered likely to interfere with reliable neurological assessment were excluded to prevent baseline functional limitations from biasing the evaluation of neurological recovery.
4. Recurrent stroke or reinfarction during the perioperative period, which would preclude accurate evaluation of the outcome attributable to the initial reperfusion therapy.
5. Incomplete data: Patients with missing clinical or imaging data, or those who failed to complete the 3-month follow-up.
6. Patients who refused participation, died before enrollment or during follow-up, or had unavailable 3-month follow-up information were excluded. Because ΔmRS required paired mRS data at admission and at 3 months, patients without complete follow-up mRS information could not be included in the primary ΔmRS-based analysis.

Considering the substantial heterogeneity among patients with acute ischemic stroke in terms of pathophysiology, responsible vessel location, treatment time window, and reperfusion modality, predefined screening criteria were applied at the study design stage to minimize the influence of these differences on the study results. First, with respect to stroke type, the present study mainly included patients with imaging-confirmed occlusion of a clearly defined responsible vessel who underwent reperfusion therapy, thereby reducing heterogeneity arising from differences in stroke subtype and underlying pathophysiological mechanisms. Second, regarding treatment timing, the interval from symptom onset to initiation of reperfusion therapy was restricted to within 24 h, and delayed thrombectomy cases were selected using uniform imaging-based criteria, ensuring relative consistency in tissue salvageability across the enrolled cohort. Third, in terms of baseline function, only patients with a pre-stroke mRS score ≤2 were included to reduce the impact of pre-existing disability on the assessment of 3-month functional outcomes. Although different reperfusion modalities were included, all patients were managed within the same stroke center using standardized workflows, which may have partly reduced the influence of treatment-pathway heterogeneity.

### 2.2 Baseline characteristics and collection of relevant variables

This study extracted patients’ general clinical data from electronic medical records, including gender, age, smoking and drinking history, and the presence of pre-existing conditions such as hypertension, diabetes, coronary heart disease, and paroxysmal or persistent atrial fibrillation. Acute-phase management information, including time from symptom onset to reperfusion therapy, was also recorded. All patients underwent standardized neurological assessment upon admission, with baseline neurological deficit severity evaluated using the NIHSS. Laboratory parameters including fingerstick blood glucose, neutrophil count, lymphocyte count, platelet count, and D-dimer were collected at admission to reflect baseline physiological and inflammatory status. All tests were performed by the hospital’s Clinical Laboratory Center using standardized automated biochemical analyzers. Initial admission laboratory results were used for analysis to ensure comparability and timeliness of indicators. For imaging, all patients underwent cranial CT or MRI prior to treatment to delineate lesion extent. The location of occluded vessels was confirmed via cranial and cervical CT angiography (CTA), or digital subtraction angiography (DSA). For anterior circulation infarcts, the ASPECTS was used; for posterior circulation infarcts, posterior circulation ASPECTS was applied when appropriate. The degree of recanalization was assessed using the Thrombolysis in Cerebral Infarction (TICI) scale. All imaging data were independently interpreted by two qualified radiologists. In cases of disagreement, a third senior attending physician reviewed the findings, and the final decision was based on consensus. Reperfusion-related variables, including the degree of recanalization assessed by the TICI scale and the time from symptom onset to reperfusion, were also recorded as part of the clinical dataset. In addition, follow-up imaging examinations (such as CT or MRI) were performed in some patients after reperfusion therapy as part of routine clinical management. However, because this study was retrospective and follow-up imaging protocols were not fully standardized across all patients, detailed imaging indicators reflecting post-reperfusion lesion evolution were not consistently available for the entire cohort. Therefore, follow-up imaging data were not systematically incorporated into the present analysis.

Treatment modality was recorded as intravenous thrombolysis alone, mechanical thrombectomy alone, or bridging therapy. Although reperfusion-related variables such as TICI grade and onset-to-reperfusion time were collected, the final model was intended as an admission-based pre-reperfusion model. Therefore, variables available at admission or before reperfusion therapy were prioritized for model construction.

### 2.3 Grouping methodology and data standardization

#### 2.3.1 Data processing

Before model development, all clinical and imaging variables underwent completeness checks, consistency verification, and logical review. Data were entered into the database using a standardized template and a double-entry procedure was applied to minimize entry errors. For variables with missing or inconsistent data, the original medical records were rechecked. Patients with unavailable key clinical, imaging, or follow-up data after rechecking were excluded from the final analysis. To reduce the impact of scale differences on parameter estimation and model convergence, selected continuous variables with substantial variation in measurement scale—including platelet count, systemic immune-inflammation index (SII), D-dimer, and onset-to-reperfusion time—were standardized using Z-scores. These preprocessing procedures were intended to improve comparability among candidate predictors and enhance model stability in subsequent regression analyses.

1. Baseline ASPECTS Score: Patients underwent plain head CT after admission, and early ischemic changes were assessed using the ASPECTS system. ASPECTS divides the middle cerebral artery territory into 10 scoring units, starting at 10 points, with one point deducted for each involved region; lower scores indicate more extensive ischemic injury. Considering that extremely low ASPECTS values often represent extensive infarct cores and may introduce instability in statistical modeling because of sparse distributions in the lower score range, this study uniformly recorded ASPECTS scores ≤6 as 6 points. This approach was intended to reduce the influence of extreme values and improve statistical stability. Similar categorization strategies have been reported in previous stroke studies investigating the prognostic value of ASPECTS in reperfusion therapy (10, 16).
2. Collateral circulation stratification: Collateral circulation grading was determined using CTA obtained after hospital admission to assess the degree of collateral blood flow perfusion to the ischemic area following occlusion of the responsible vessel. Based on prior literature and relevant clinical consensus, this study defined grades 0–1 as poor collateral circulation and grades 2–3 as good collateral circulation (17). This grading method exhibits high radiographic reproducibility and clinical relevance, and has been widely used for collateral circulation assessment and prognostic analysis in AIS patients.
3. Systemic Immune-Inflammation Index (SII): To avoid redundancy among correlated inflammatory variables, this study selected the systemic immune-inflammation index (SII) as a composite indicator reflecting the severity of the patient’s inflammatory response. SII integrates platelet count, neutrophil count, and lymphocyte count into a single parameter and has been widely used as a comprehensive marker of inflammatory and immune status in cerebrovascular research. Compared with including multiple correlated inflammatory variables simultaneously, the use of SII may help reduce redundancy among predictors and improve model stability (18).
4. Time from Onset to Recanalization: This refers to the interval from the onset of stroke symptoms to the achievement of effective vascular recanalization. For patients whose exact onset time could not be determined, such as wake-up stroke, the “last known well” time was used as the surrogate onset time, which is a commonly accepted approach in stroke research and clinical practice. For patients receiving intravenous thrombolysis alone, recanalization time was defined as the time when imaging confirmed restoration of blood flow after thrombolytic treatment. For patients undergoing mechanical thrombectomy, the time point when TICI 2b–3 reperfusion was first achieved during angiography was considered the recanalization time (19). Although these definitions correspond to slightly different procedural events, they all represent clinically meaningful time points reflecting effective reperfusion.
5. Age-adjusted Charlson Comorbidity Index (aCCI): The aCCI is a comprehensive metric assessing systemic health burden and long-term mortality risk. It employs weighted scoring for multiple chronic conditions and age factors to reflect an individual’s overall physiological reserve and metabolic homeostasis. Higher aCCI scores indicate greater comorbidity, elevated systemic inflammation, and poorer organ functional reserve.
6. To avoid parameter estimation bias due to inconsistent variable scales, this study standardized certain metrics—including platelet count, SII, D-dimer, and time from onset to reperfusion—using Z-score normalization. This process centered each variable and scaled it by standard deviation. This method ensures all variables participate in the analysis at the same magnitude, enhancing model convergence and comparability.

#### 2.3.2 ΔmRS calculation and grouping method

In this study, mRS was assessed at admission and at 3 months after treatment. The admission mRS score reflected acute functional status before or at the initiation of reperfusion therapy. It was different from the pre-stroke mRS score, which was used only as an eligibility criterion. ΔmRS was calculated as admission mRS minus 3-month mRS. A positive value indicated functional improvement, and a larger value indicated greater improvement. A value of zero or less indicated no improvement or functional deterioration. Therefore, ΔmRS was treated as a directional indicator of functional improvement rather than an absolute difference. Patients with ΔmRS ≥ 2 were classified as having significant functional improvement, whereas those with ΔmRS < 2 were classified as having non-significant improvement or poor functional recovery. This grouping was used for risk stratification and model development in the present study.

### 2.4 Statistical methods

All statistical analyses and model development were performed using R software (version 4.3.1). Continuous variables were first assessed for normality. Normally distributed variables are presented as mean ± standard deviation (SD) and compared using independent-samples t tests, while non-normally distributed variables are expressed as median (interquartile range, IQR) and compared using the Mann–Whitney U test. Categorical variables are presented as counts and percentages and were compared using the chi-square test. To develop and validate the prediction model, all eligible patients were randomly divided into a training cohort and a validation cohort at a ratio of 7:3. The number and proportion of poor functional recovery events (ΔmRS < 2) were reported in the overall cohort, training cohort, and validation cohort. This split-sample approach was used as an internal validation strategy to assess the robustness and reproducibility of the model.

Candidate variables were selected based on a combination of clinical relevance, prior literature, and data availability. Only variables available at admission or before reperfusion therapy were considered for the final admission-based model. Procedural or post-reperfusion variables were not included in the final model. Because multicollinearity may exist among clinical and laboratory variables and the number of candidate predictors was relatively large, LASSO regression was applied as a penalized method to shrink coefficients and select variables, thereby reducing the potential impact of multicollinearity on model stability. The optimal penalty parameter (*λ*) was determined using 10-fold cross-validation to reduce overfitting and improve the stability of variable selection. Variables with non-zero coefficients under the selected λ were retained for subsequent analysis. After LASSO-based variable selection, multicollinearity among the retained predictors was further assessed using the variance inflation factor (VIF). A VIF value <5 was considered to indicate no serious multicollinearity. The final prediction model was constructed based on the variables retained after LASSO selection using multivariable logistic regression, with all selected predictors simultaneously entered into the model to estimate their independent effects. This modeling strategy allows mutual adjustment among predictors and helps reduce the potential influence of confounding factors. A nomogram was subsequently constructed based on the final independent predictors to provide an individualized risk estimation tool.

Model performance was evaluated in terms of discrimination, calibration, and clinical utility. Discrimination was assessed using receiver operating characteristic (ROC) curves and the area under the ROC curve (AUC). In addition, sensitivity and specificity at clinically relevant cutoff points were considered to further evaluate the discriminative performance of the model. Calibration was assessed using calibration curves to examine the agreement between predicted probabilities and observed outcomes. Clinical usefulness was evaluated using decision curve analysis (DCA) to estimate the net clinical benefit across a range of threshold probabilities.

**Figure 2:**
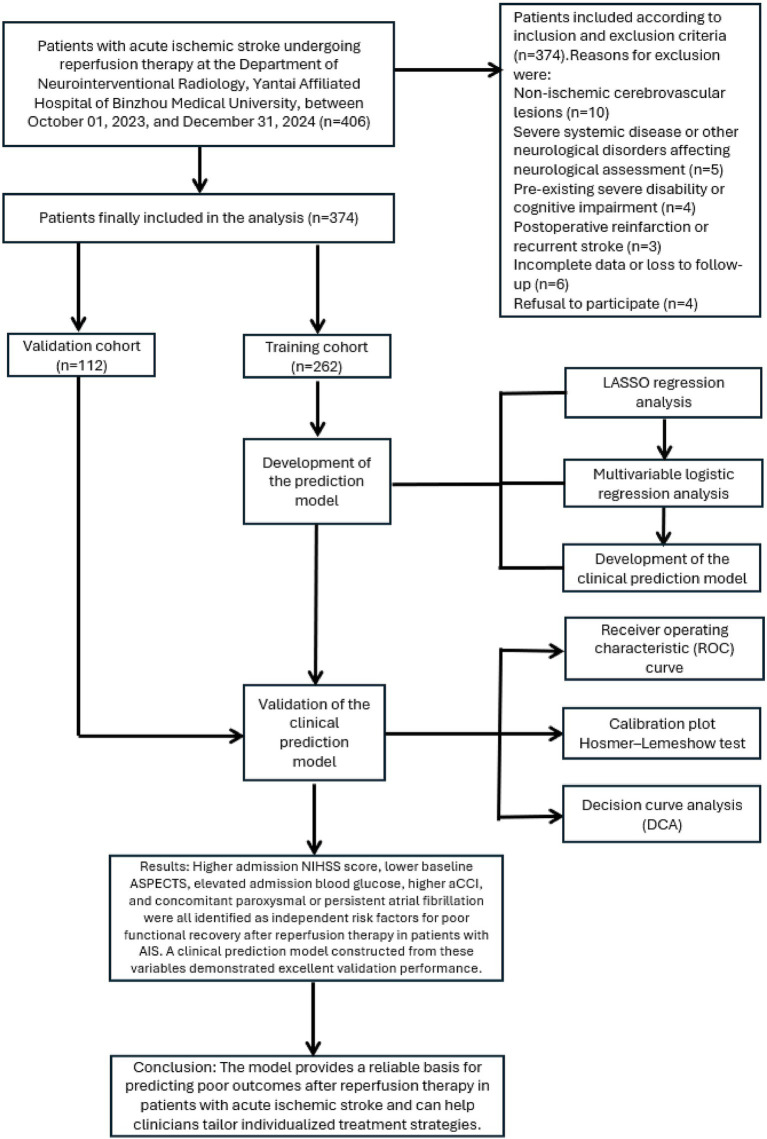
Prediction model flowchart.

All model performance metrics were evaluated in both the training and validation cohorts to ensure internal validation. All statistical tests were two-sided, and *p* < 0.05 was considered statistically significant. The overall process of this study, including the main steps of research design, data collection, model construction, and validation, is shown in Figure 2.

## 3 Results

### 3.1 Baseline demographic and clinical characteristics of patients

This study initially enrolled 406 AIS patients, with 374 ultimately included based on the predefined inclusion and exclusion criteria. Exclusion reasons included non-ischemic cerebrovascular lesions (*n* = 10), severe systemic diseases or other neurological disorders affecting neurological assessment (*n* = 5), prior severe disability or cognitive impairment (*n* = 4), postoperative reinfarction or recurrent stroke (*n* = 3), incomplete data or missing follow-up (*n* = 6), and refusal to participate or death prior to enrollment (*n* = 4). All excluded cases were removed strictly according to the predefined criteria rather than outcome-related factors. Among the excluded patients, 6 cases were excluded because of incomplete data or missing follow-up, accounting for 1.5% of the initially screened cohort. Because the number of excluded cases was relatively small and the exclusion was based on predefined criteria rather than outcome-related factors, the potential impact on the representativeness of the study population is likely to be limited.

To support model development and internal validation, computerized random allocation was used to divide eligible patients into a training cohort (*n* = 262) and a validation cohort (*n* = 112) at a 7:3 ratio. Poor functional recovery, defined as ΔmRS < 2, occurred in 124 of 374 patients (33%). In the training cohort, 90 of 262 patients (34%) had poor functional recovery, and in the validation cohort, 34 of 112 patients (30%) had poor functional recovery. The training cohort was used to construct the clinical prediction model, while the validation cohort served for internal validation. Baseline demographic and clinical characteristics were compared between the training and validation cohorts. No statistically significant differences were observed in age, sex, comorbidities, neurological function scores, or laboratory indicators, suggesting that the two cohorts were generally comparable. Regarding treatment modality, 243 patients (65%) received intravenous thrombolysis alone, 56 patients (15%) underwent mechanical thrombectomy alone, and 75 patients (20%) received bridging therapy. The distribution of outcome events and treatment modality is shown in Table 1.

**Table 1:** Patient demographic and baseline characteristics.

Variable		Cohort		*p*-value^b^
		Training cohort *N* = 262^a^	Validation cohort *N* = 112^a^	
Age		69 (61, 75)	69 (63, 75)	0.635
Gender	Male	171 (65.3%)	67 (59.8%)	0.316
	Female	91 (34.7%)	45 (40.2%)	
Admission NIHSS score		4.0 (2.0, 9.0)	4.0 (3.0, 6.0)	0.494
Baseline ASPECTS score		8.00 (7.00, 9.00)	8.00 (7.00, 9.00)	0.307
Platelet		−0.09 (−0.83, 0.39)	−0.03 (−0.69, 0.39)	0.409
Time from onset to thrombolysis		−0.27 (−0.50, 0.19)	−0.26 (−0.47, 0.08)	0.972
D-dimer		0.70 (0.46, 1.28)	0.68 (0.49, 0.99)	0.680
Systemic Inflammation Index		−0.43 (−0.65, 0.31)	−0.40 (−0.63, 0.35)	0.815
Admission Blood Glucose		7.04 (6.10, 8.40)	6.97 (6.12, 8.60)	0.825
aCCI		6.00 (3.00, 8.00)	6.00 (3.00, 7.00)	0.683
Atrial Fibrillation	No	199 (76.0%)	92 (82.1%)	0.187
	Yes	63 (24.0%)	20 (17.9%)	
Smoking	No	87 (33.2%)	46 (41.1%)	0.146
	Yes	175 (66.8%)	66 (58.9%)	
Hypertension	No	94 (35.9%)	37 (33.0%)	0.598
	Yes	168 (64.1%)	75 (67.0%)	
Alcohol consumption	No	196 (74.8%)	88 (78.6%)	0.436
	Yes	66 (25.2%)	24 (21.4%)	
Collateral circulation grading	Good	126 (48.1%)	65 (58.0%)	0.078
	Poor	136 (51.9%)	47 (42.0%)	
Poor functional recovery, *n* (%)		90(34.3%)	34(30.3%)	0.452
Significant functional improvement, *n* (%)		172(65.7%)	78(69.7%)	
Treatment modality, *n* (%)	IVT alone	170 (64.9%)	73 (65.2%)	0.991
	MT alone	39 (14.9%)	17 (15.2%)	
	Bridging therapy	53 (20.2%)	22 (19.6%)	

a Median (Q1, Q3); *n* (%).

b Wilcoxon rank sum test; Pearson’s Chi-squared test.

### 3.2 LASSO regression variable selection results

**Figure 3:**
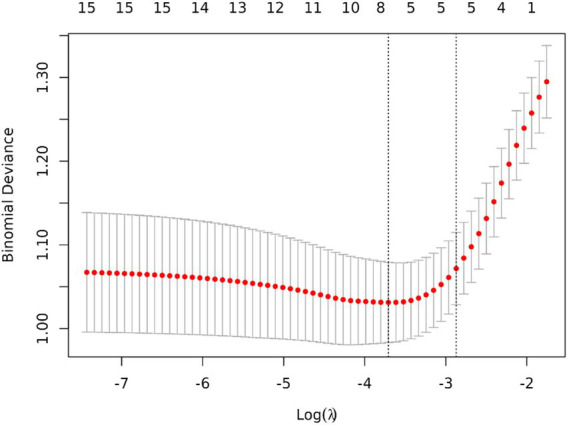
LASSO regression cross-validation plot.

**Figure 4:**
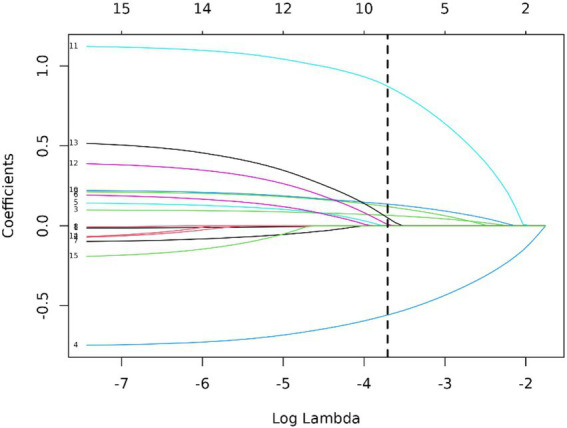
LASSO regression coefficient path diagram.

In the training cohort, LASSO regression was performed using the ΔmRS-based outcome grouping as the dependent variable and all candidate clinical, laboratory, and imaging indicators as independent variables for dimensionality reduction and variable selection. The penalty parameter (*λ*) was determined using 10-fold cross-validation, and the final model was selected with consideration of model parsimony, stability, and clinical interpretability. Ultimately, seven variables with non-zero coefficients were retained, including admission NIHSS score, admission blood glucose level, baseline ASPECTS score, hypertension, atrial fibrillation, aCCI, and smoking history, and were subsequently entered into the multivariable logistic regression model. This step was intended to identify predictors with stronger associations with the outcome from a relatively large candidate set and to partly reduce the influence of redundant information on subsequent model development. The corresponding 10-fold cross-validation plot and LASSO coefficient path plot are presented in Figures 3, 4, respectively.

**Figure 5:**
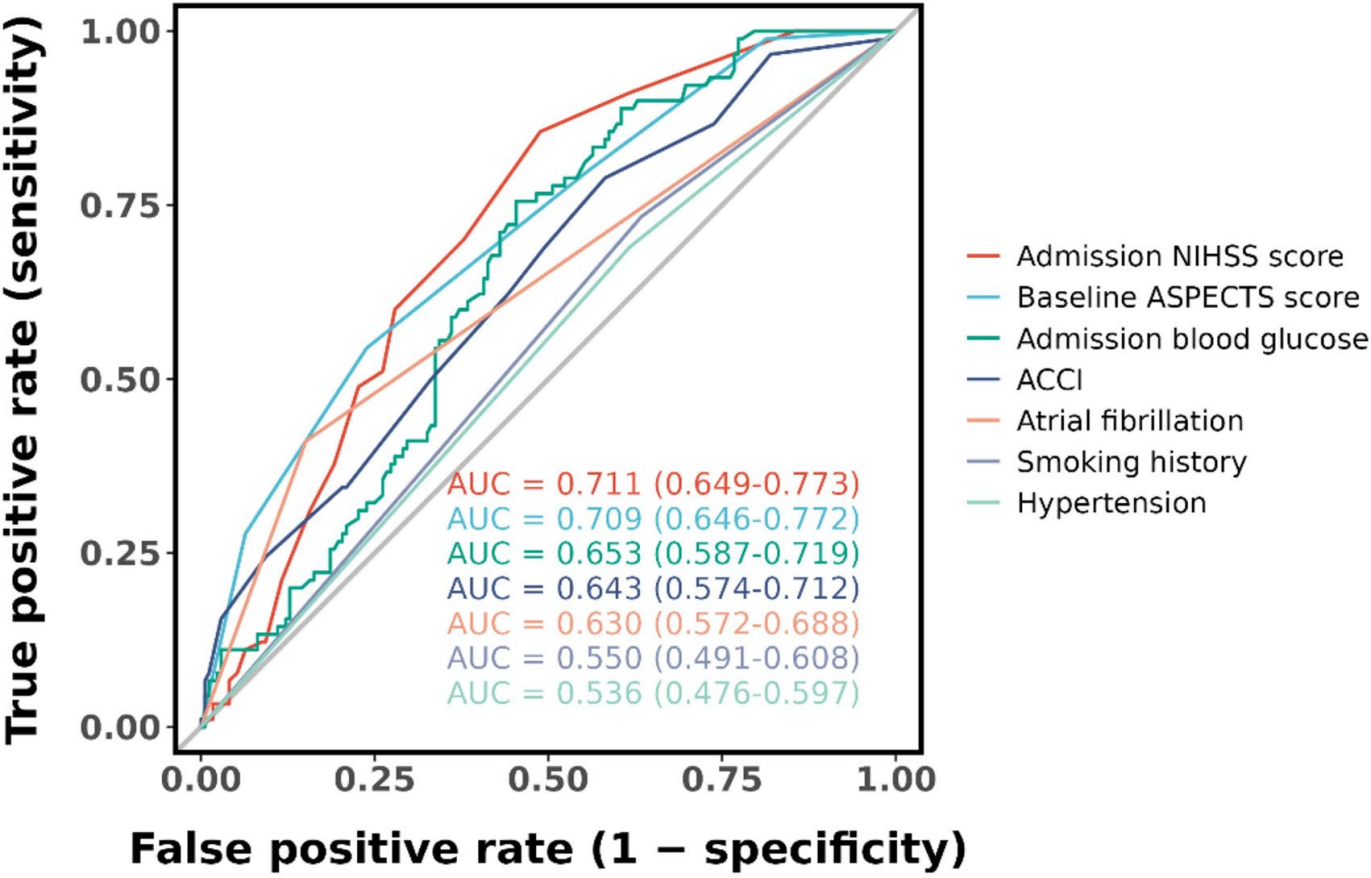
ROC curve analysis of seven candidate diagnostic indicators.

This study further conducted multivariable logistic regression analysis on the key variables selected by LASSO regression. ROC curves were plotted for each potential risk factor to assess their discriminatory ability and predictive performance for poor prognosis. Exploratory ROC analysis was further performed for the variables retained by LASSO regression to describe their individual discriminatory ability. However, these variables were ultimately evaluated within the multivariable prediction model rather than interpreted as stand-alone predictors (Figure 5).

### 3.3 Multivariable logistic regression analysis results

To investigate whether the seven variables selected by LASSO regression analysis represent independent risk factors for poor recovery after AIS reperfusion, this study further employed multivariable logistic regression analysis to adjust for potential confounding factors. Multicollinearity diagnostics showed that the VIF values of all variables included in the final multivariable model were below 5, indicating no serious multicollinearity among the retained predictors. Results showed that admission NIHSS score (OR = 1.10, 95% CI: 1.03–1.17, *p* = 0.003), baseline ASPECTS score (OR = 0.48, 95% CI: 0.37–0.64, *p* < 0.001), and admission blood glucose level (OR = 1.24, 95% CI: 1.04–1.47, *p* = 0.019), aCCI (OR = 1.20, 95% CI: 1.08–1.34, *p* < 0.001), and concomitant paroxysmal or persistent atrial fibrillation (OR = 3.34, 95% CI: 1.66–6.73, *p* < 0.001) were independent predictors of poor functional recovery after AIS reperfusion therapy, as detailed in Table 2. The odds ratios (OR), and corresponding 95% confidence intervals of all variables included in the final model are presented in Table 2, allowing full transparency of the model structure. The regression coefficients (*β*) were exponentiated and reported as odds ratios (OR) with 95% confidence intervals to facilitate clinical interpretation.

**Table 2:** Multivariable logistic regression results for the training cohort.

Characteristic		N	Event N	OR	95% CI	*p*-value
Admission NIHSS Score		262	90	1.10	1.03, 1.17	0.003
Baseline ASPECTS score		262	90	0.48	0.37, 0.64	<0.001
Admission blood glucose		262	90	1.24	1.04, 1.47	0.019
aCCI		262	90	1.20	1.08, 1.34	<0.001
Atrial fibrillation	No	199	53	—	—	
	Yes	63	37	3.34	1.66, 6.73	<0.001
Smoking	No	87	24	—	—	
	Yes	175	66	1.40	0.70, 2.77	0.339
Hypertension	No	94	28	—	—	
	Yes	168	62	1.56	0.81, 3.04	0.187

CI, Confidence Interval; OR, Odds Ratio.

**Figure 6:**
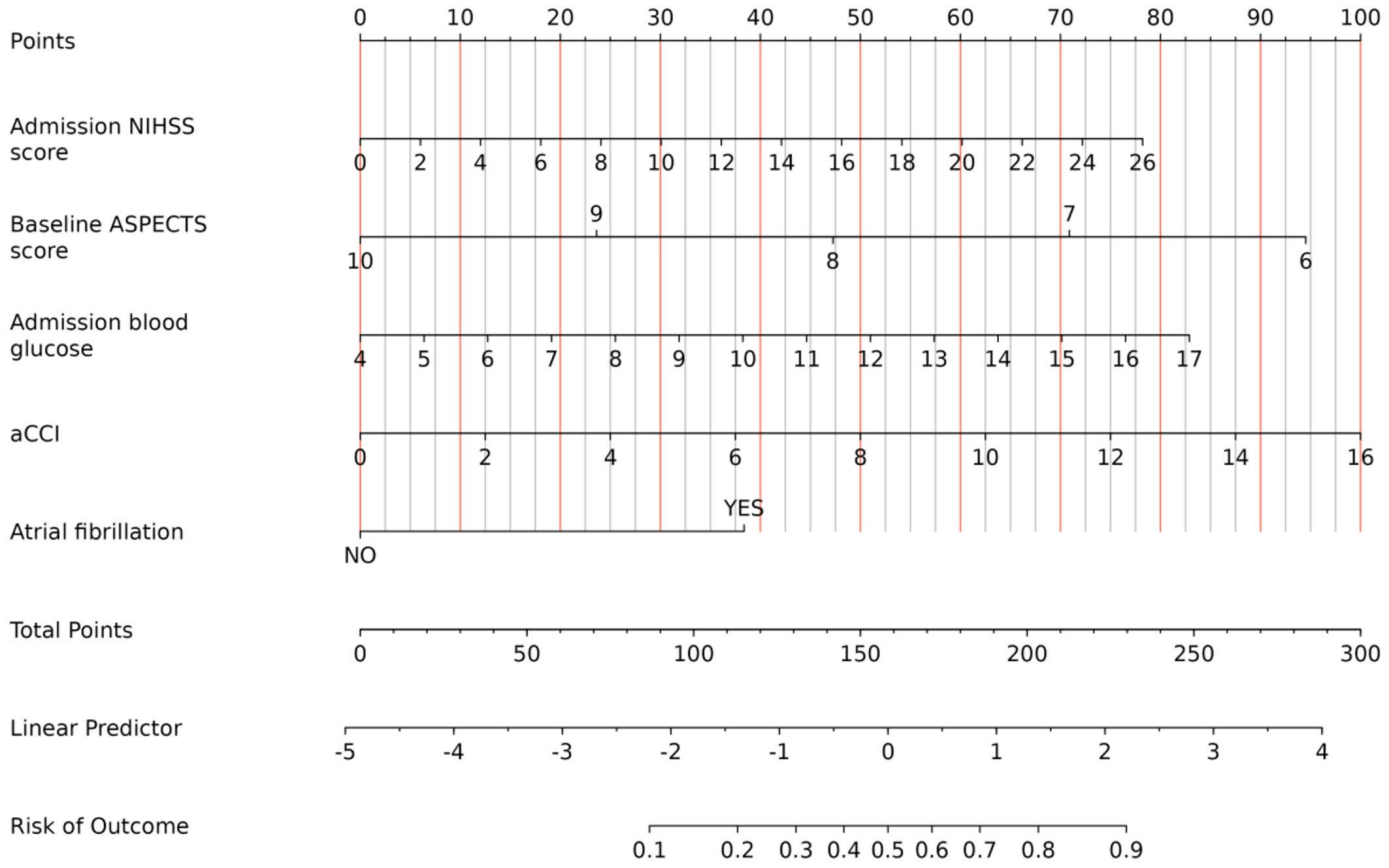
Nomogram for predicting poor functional recovery after reperfusion therapy.

Based on these admission-based predictors, a pre-reperfusion clinical prediction model was constructed and presented as a nomogram (Figure 6). This model can be used to assess the risk of poor functional recovery following reperfusion therapy in AIS patients.

### 3.4 Validation of the predictive model

#### 3.4.1 Analysis of model accuracy and robustness

**Figure 7:**
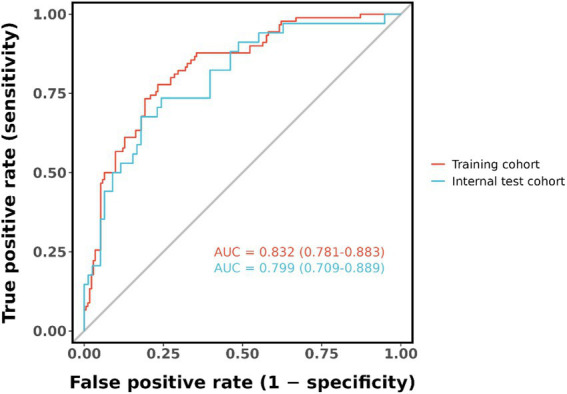
ROC curves for the prediction model.

The model’s discriminatory ability and robustness were evaluated by calculating the ROC curves and AUC values for both the training and validation cohorts. To assess discriminatory capability, ROC curves and AUC values were computed separately for the training and validation cohorts, with overlap between curves compared to observe consistency and stability across samples (20). The AUC was 0.832 (95% CI 0.781–0.883) in the training cohort and 0.799 (95% CI 0.709–0.889) in the validation cohort (Figure 7). These results suggest acceptable discrimination and internal reproducibility in the present cohort. However, because the validation cohort was obtained from a random split of the same single-center dataset, this should be regarded as internal validation. External validation is still needed before the model can be applied more broadly.

#### 3.4.2 Model calibration curve assessment

**Figure 8:**
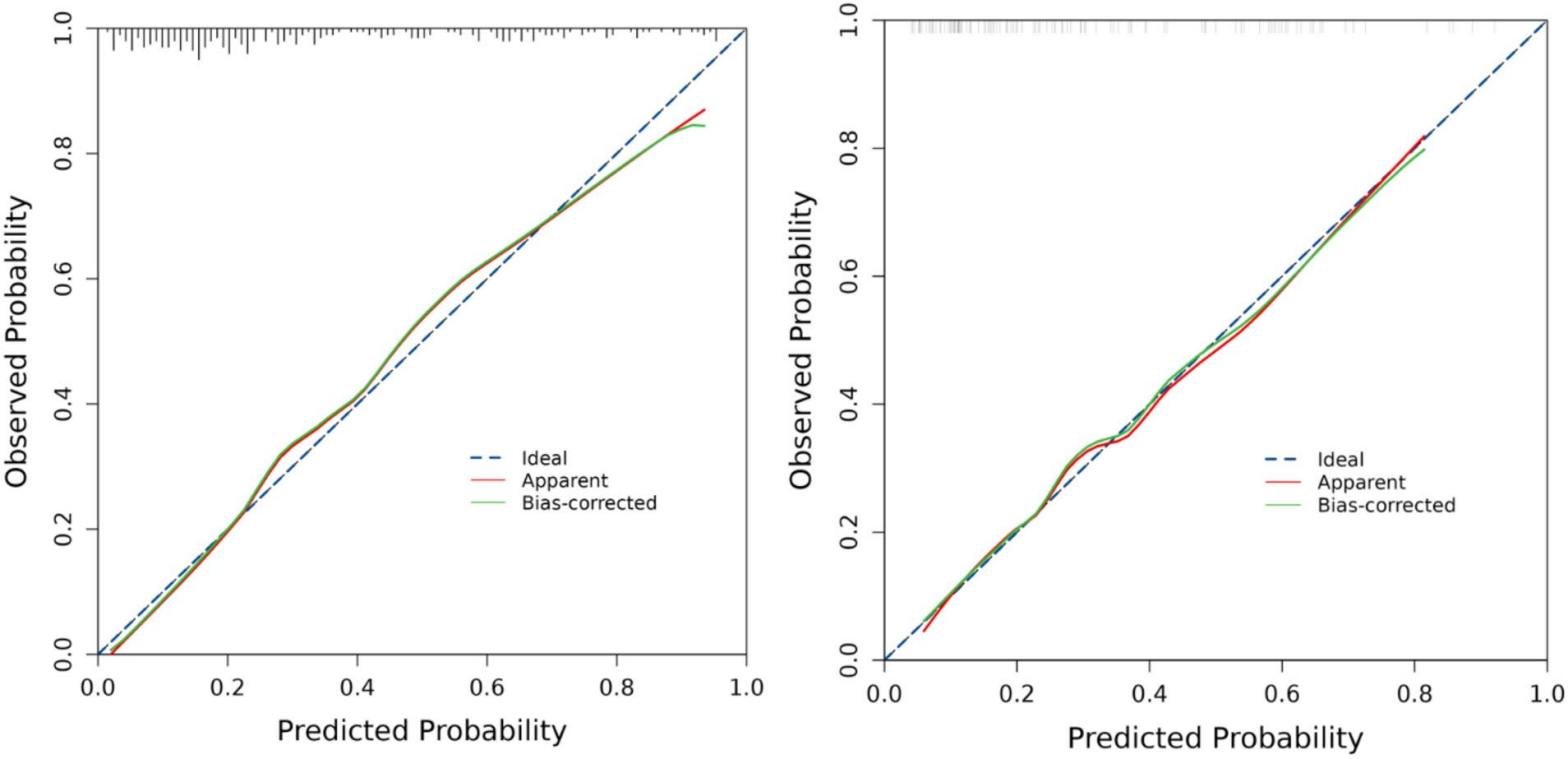
Calibration curves for the regression model on the training and validation cohorts.

To further assess model calibration, calibration curves were generated for the training and validation cohorts using the riskRegression package in R (Figure 8). Calibration analysis was performed to evaluate the agreement between the predicted probabilities and the observed event rates. In general, a calibration curve closer to the 45° reference line indicates better agreement between predicted and observed probabilities. A curve located above the reference line suggests risk underestimation, whereas a curve located below the reference line indicates risk overestimation. As shown in Figure 8, the calibration curves of both the training and validation cohorts were generally close to the ideal reference line, indicating good agreement between the predicted probabilities and the observed outcomes (21). These findings suggest that the model showed favorable calibration performance in both datasets, and that the predicted probabilities of ΔmRS improvement were broadly consistent with the actual observed event rates.

#### 3.4.3 Decision curve analysis of the model

**Figure 9:**
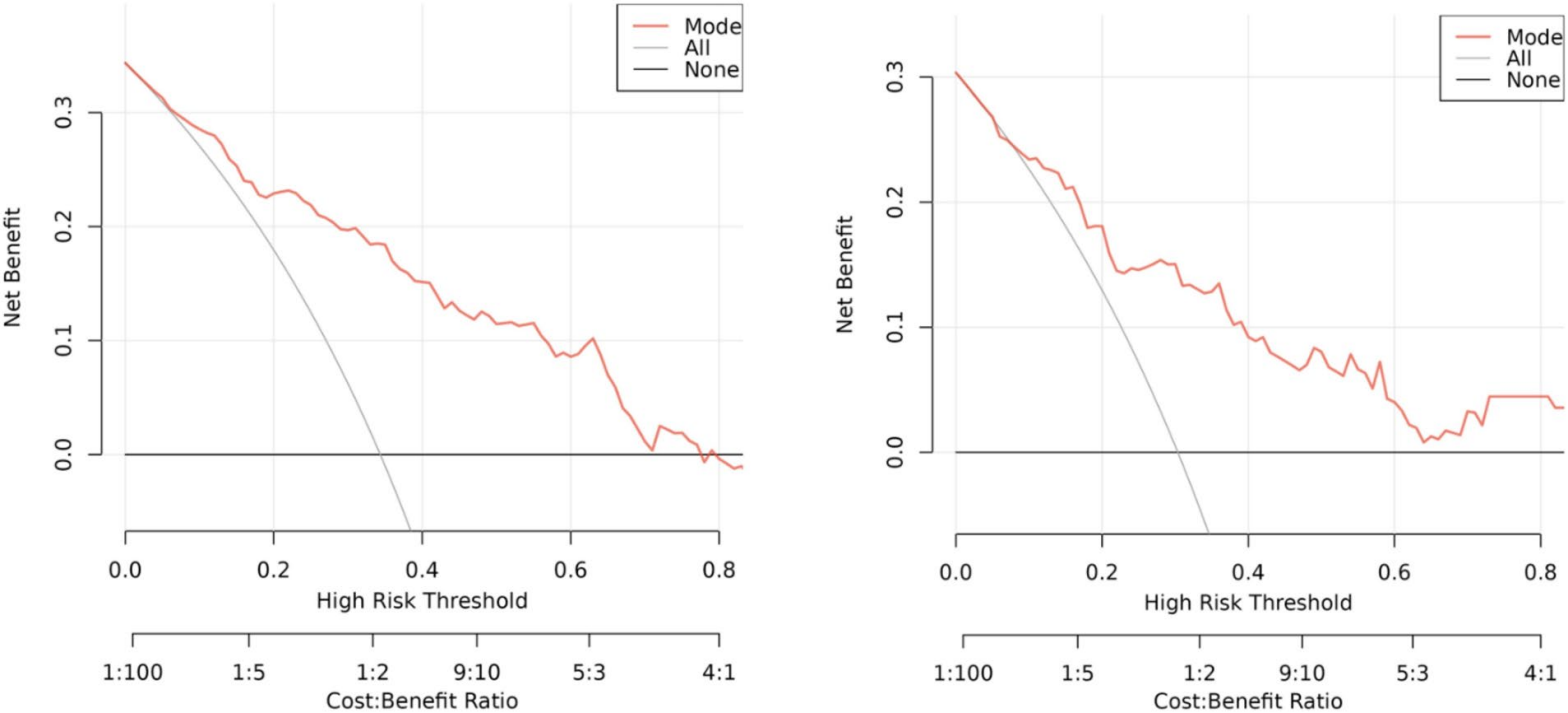
Decision curve analysis of the prediction model in the training and validation cohorts.

To evaluate the clinical utility of a prediction model, DCA provides an intuitive method for assessing its potential value in clinical decision-making across a range of risk thresholds. Although conventional evaluation metrics can reflect the statistical performance of a model, they do not directly indicate whether the model can improve real-world clinical decision-making. By incorporating the balance between the benefits of correctly identifying high-risk patients and the harms associated with unnecessary intervention in low-risk patients, DCA estimates the net clinical benefit of the model under different threshold probabilities. This approach therefore offers a practical framework for determining whether the use of a prediction model may support individualized intervention strategies in specific clinical settings. In addition, comparison of the model with default strategies, such as treating all patients or treating none, helps define the threshold ranges in which the model provides added clinical value (22). The present results suggested that the model may provide net benefit within certain threshold ranges. However, this finding should be interpreted as exploratory because the model was evaluated only by internal validation in a single-center cohort (Figure 9).

## 4 Discussion

This study systematically investigated factors influencing poor functional recovery after reperfusion therapy in AIS patients using multidimensional clinical, laboratory, and imaging data, and developed an individualized prognostic prediction model. Results indicate that admission NIHSS score, baseline ASPECTS score, admission blood glucose level, aCCI value, and atrial fibrillation are independent predictors of poor functional recovery after reperfusion therapy. Regarding model performance, variables selected via LASSO regression were integrated into a clinical prediction model through multivariable logistic regression modeling. Overall, these findings are consistent with previous studies, while further extending prognostic modeling within a ΔmRS-based dynamic outcome framework. The model showed acceptable discrimination in the training and internal validation cohorts, and the calibration and decision curve analyses suggested possible clinical usefulness within the present dataset. Because all predictors retained in the final model were available at admission or before reperfusion therapy, the model should be interpreted as an admission-based pre-reperfusion risk stratification tool. Its performance in other centers or in specific treatment-modality subgroups still requires further validation.

In the present study, ΔmRS was introduced as a dynamic outcome indicator not with the intention of replacing established endpoints such as the conventional 90-day mRS, but rather to provide a complementary perspective on post-treatment recovery from the viewpoint of functional improvement magnitude. The traditional 90-day mRS has strong clinical interpretability and remains one of the most widely accepted outcome measures in stroke research, as it effectively reflects the patient’s overall functional status at the follow-up endpoint. It therefore continues to serve as a fundamental outcome measure in prognostic studies of stroke. However, the 90-day mRS is essentially a fixed endpoint measure and provides only limited information about the recovery process or the magnitude of improvement achieved relative to baseline. In contrast, ΔmRS places greater emphasis on functional change itself and may therefore offer additional information regarding individualized treatment benefit, particularly in patients with different levels of baseline neurological impairment (9, 23).

It should also be acknowledged that ΔmRS is not currently a universally established primary endpoint in stroke prognostic research, and its clinical applicability boundaries, optimal stratification threshold, and cross-population stability still require further validation. In addition, because the mRS is an ordinal functional scale, ΔmRS may still be influenced by inter-rater variability, differences in baseline interpretation, and variability in rehabilitation adherence. Accordingly, the present study considers ΔmRS as a supplementary and exploratory dynamic outcome measure intended to enrich, rather than replace, the conventional fixed-endpoint evaluation framework. Future studies may further integrate the traditional 90-day mRS, early neurological improvement measures, and imaging-based outcomes to achieve a more comprehensive assessment of the stroke recovery process. Although studies specifically using ΔmRS as a primary prognostic endpoint remain limited, prior research in stroke recovery has increasingly emphasized the importance of functional change and recovery trajectories rather than static endpoints alone (24, 25). This conceptual framework provides indirect support for the use of ΔmRS as a complementary indicator for evaluating treatment benefit and recovery dynamics.

### 4.1 NIHSS score at admission

The admission NIHSS score is a standardized scale for assessing the severity of neurological deficits in AIS patients, comprehensively reflecting multidimensional neurological function including motor, language, consciousness, and sensory status. A higher score indicates more severe brain injury at admission, larger infarct core volume, and less salvageable ischemic penumbra, thereby limiting neurological recovery following reperfusion therapy (26). The NIHSS score is a widely used indicator reflecting the severity of neurological deficits in the acute phase of stroke. In this study, higher admission NIHSS scores were significantly associated with an increased risk of poor functional recovery, which is consistent with previous reports (27). Specifically, each 1-point increase in baseline NIHSS score was associated with approximately a 10% higher risk of poor functional recovery after reperfusion therapy (OR = 1.10, 95% CI: 1.03–1.17, *p* = 0.003), indicating that the severity of neurological deficits is a core determinant of reperfusion efficacy. Mechanistically, a higher NIHSS score generally reflects more extensive brain injury and more severe neurological impairment, thereby limiting the potential for functional recovery. In addition, patients with higher NIHSS scores are often associated with more complex hemodynamic disturbances and a reduced benefit from reperfusion therapy, which may further contribute to poorer long-term outcomes. Studies by Goyal et al. showed that even after successful recanalization, patients with an admission NIHSS score ≥15 had more than twice the risk of a 3-month mRS score >3 (28). Other studies further indicated that higher admission NIHSS scores were closely associated with poor collateral circulation, recanalization failure, and an increased risk of hemorrhagic transformation (29, 30). Wang et al. also identified admission NIHSS score as an independent predictor of reperfusion success and early neurological improvement (31). These findings suggest that a high admission NIHSS score may reflect more severe brain injury, a larger ischemic burden, and a lower likelihood of tissue salvage, thereby contributing to poor prognosis after recanalization. Accordingly, greater perioperative caution is warranted in these patients (32). However, the relationship between NIHSS score and prognosis is not strictly linear, and a high NIHSS score does not necessarily indicate irreversible neurological injury. In some cases, particularly when rapid reperfusion is achieved within the ultra-early time window, patients with high baseline NIHSS scores may still experience substantial neurological recovery. This suggests that timely reperfusion may partially offset the adverse prognostic impact of severe baseline deficits, possibly through the preservation of the ischemic penumbra before irreversible infarction occurs (33). Overall, admission NIHSS score reflects not only the severity of neurological deficits but also, to some extent, the status of cerebral perfusion and tissue injury. It remains an important predictor of functional outcome after reperfusion therapy in patients with acute ischemic stroke.

### 4.2 Baseline ASPECTS score

The baseline ASPECTS score is a semi-quantitative measure based on non-contrast cranial CT for evaluating early ischemic changes in the anterior circulation. It is commonly used to assess the extent of early cerebral ischemia and can indirectly reflect infarct core burden and the degree of potentially reversible tissue injury. The score ranges from 0 to 10, with lower scores indicating more extensive early ischemic involvement and a greater proportion of irreversible damage. This is generally associated with a larger infarct core, a smaller salvageable penumbra, and greater disruption of blood–brain barrier integrity. Barber et al. (34) reported that ASPECTS can be used to quantify the extent of early cerebral infarction, guide reperfusion treatment decisions, and predict neurological recovery and complication risk after reperfusion therapy, thereby providing important clinical value. At present, ASPECTS is regarded not only as an approximate indicator of ischemic lesion extent, but also as an important marker of the potential upper limit of reperfusion benefit, as larger infarct cores with less salvageable tissue are generally associated with more limited benefit from reperfusion. This study found a significant negative correlation between baseline ASPECTS score and functional recovery after reperfusion (OR = 0.48, 95% CI: 0.37–0.64, *p* < 0.001). Results indicate that a lower baseline ASPECTS score and greater extent of early ischemic injury may increase the risk of poor neurological recovery in patients. Goyal et al. found that among patients with acute ischemic stroke receiving standard reperfusion therapy, those with baseline ASPECTS scores ≥7 derived greater benefit from recanalization, whereas those with baseline ASPECTS scores ≤5 showed more limited improvement in prognosis (28). This suggests that in patients with lower ASPECTS scores, the potential benefit of reperfusion may be restricted by a larger infarct core burden. Campbell et al. further reported that baseline ASPECTS score was an independent predictor of post-thrombectomy infarct volume, hemorrhagic transformation risk, and 90-day functional outcome (10). In addition to reflecting the extent of ischemic injury, ASPECTS may also, to some extent, indicate differences in collateral circulation status and tissue viability (35, 36). Therefore, in patients with low baseline ASPECTS scores, further preoperative evaluation with CT perfusion or diffusion-weighted/perfusion MRI may be helpful for assessing the extent of salvageable tissue. On this basis, the indication for reperfusion therapy should be selected more cautiously, and greater attention should be paid to reperfusion management during treatment to reduce the risk of hemorrhagic complications. However, a low ASPECTS score does not necessarily indicate absence of benefit. Huo et al. reported that in the extended time window beyond 6 h after stroke onset, some patients with low ASPECTS scores may still benefit from reperfusion therapy if substantial perfusion mismatch or penumbral tissue is preserved (37). This finding suggests that, in selected cases, imaging-based tissue evaluation may be more informative than time window alone for treatment decision-making. This phenomenon may be attributable to better collateral circulation, slower ischemic progression, and the persistence of salvageable tissue despite a relatively large ischemic core on imaging. It also suggests that reliance on ASPECTS alone may result in underestimation or overestimation of treatment benefit at the individual level. Therefore, combining ASPECTS with perfusion parameters, collateral circulation grading, and clinical severity indicators such as the NIHSS score may allow more accurate patient selection and more individualized management strategies. Overall, baseline ASPECTS scores serve as critical radiographic indicators for post-reperfusion prognosis assessment, with lower scores indicating more severe structural damage and limited functional recovery potential. Future research should integrate perfusion quantitative parameters with radiomic features to establish multimodal image fusion models, enabling precise prediction and individualized assessment of reperfusion therapy risks versus benefits.

In addition, because extremely low ASPECTS values often correspond to extensive infarct cores with limited potential for tissue salvage, grouping these values may reduce model instability caused by sparse distributions in the lower score range, while preserving the broad prognostic distinction between patients with limited versus extensive ischemic injury.

### 4.3 Admission blood glucose levels

Admission blood glucose is an important biochemical indicator reflecting the acute metabolic status and stress response in patients with acute ischemic stroke. Acute hyperglycemia may result from pre-existing abnormalities in glucose metabolism or from the stress response induced by stroke, including sympathetic activation, enhanced gluconeogenesis, and peripheral insulin resistance mediated by elevated catecholamines and cortisol. In this study, higher admission blood glucose was independently associated with an increased risk of poor functional recovery after reperfusion (OR = 1.24, 95% CI: 1.04–1.47, *p* = 0.019), suggesting that hyperglycemia is an important prognostic risk factor and a useful parameter for clinical risk stratification and management. Potential mechanisms include increased oxidative stress, disruption of the blood–brain barrier, and enhanced inflammatory responses under hyperglycemic conditions, all of which may aggravate reperfusion injury and impair neurological recovery (38). In addition, hyperglycemia may adversely affect microvascular perfusion and reduce the effectiveness of reperfusion therapy, thereby contributing to unfavorable outcomes. Shao et al. reported that hyperglycemia may aggravate ischemic brain injury and impair reperfusion through protein kinase C pathway activation and subsequent microvascular endothelial damage, thereby increasing the risk of poor functional recovery after reperfusion therapy in patients with acute ischemic stroke (39). Qian et al. further found that patients with admission blood glucose levels >8.0 mmol/L had a significantly higher proportion of 90-day mRS scores >2 after reperfusion therapy (40). In addition, Yaghi et al. showed that elevated admission blood glucose was closely associated with increased mortality, larger infarct volume, and failed reperfusion after stroke (41). Taken together, these findings suggest that acute hyperglycemia is not only a marker of metabolic disturbance, but also reflects impaired energy metabolism in ischemic brain tissue and excessive inflammatory activation. Studies by Ferrari et al. suggest that mild hyperglycemia in the ultra-early stage of stroke may represent a stress-related compensatory response aimed at maintaining energy and glucose metabolism. However, whether this response confers neuroprotective effects remains uncertain. In clinical practice, the potential adverse effects of hyperglycemia should be balanced against the risks associated with overly aggressive glucose lowering and subsequent hypoglycemia (42). Therefore, in patients with elevated admission blood glucose, close dynamic glucose monitoring should be performed before and after reperfusion therapy to minimize excessive fluctuations. In addition, early and appropriate glucose management may help reduce reperfusion-related injury. Overall, admission blood glucose is an independent predictor of functional outcome after reperfusion therapy in AIS, and maintaining stable, moderately controlled glycaemia is likely to be important for optimizing prognosis. Future research should focus on developing comprehensive predictive models that integrate indices of glycaemic variability, insulin resistance and glucose-metabolism–related inflammatory markers to enable more precise metabolic interventions and personalized management.

### 4.4 aCCI index

The aCCI is a composite measure of comorbidity burden that incorporates weighted chronic conditions together with age adjustment. It is widely used to assess overall health status and long-term mortality risk. Higher aCCI scores indicate a greater burden of comorbidity, reduced physiological reserve, and impaired homeostatic capacity (43). In this study, aCCI was significantly associated with poor functional recovery after reperfusion (OR = 1.20, 95% CI 1.08–1.34,*p* < 0.001), suggesting that increasing comorbidity burden and diminished systemic reserve substantially limit the potential for neurological improvement and increase the likelihood of an unfavourable prognosis. Culaj et al. (44) reported that patients with an aCCI score ≥5 had a significantly lower rate of functional independence at 3 months after reperfusion therapy. Idicula et al. (45) further found that a higher comorbidity burden was associated with slower recovery and a greater risk of recurrence after stroke, suggesting that comorbidity assessment may help predict the pace of functional recovery and long-term prognosis. Consistent with previous studies, our findings support the aCCI score as a useful clinical indicator linking systemic health burden to recanalization outcomes. Higher aCCI scores were associated with a lower likelihood of functional recovery. These findings suggest that greater attention should be given to perioperative systemic management in patients with elevated aCCI scores. Before treatment, rapid assessment of cardiopulmonary and renal function, infection risk, nutritional status, and prior medication use may help optimize the management of underlying conditions without delaying reperfusion. During treatment, emphasis should be placed on maintaining hemodynamic stability. After treatment, closer attention to blood pressure, glucose and lipid metabolism, nutritional support, and early prevention of complications such as infection and deep vein thrombosis may be warranted. When necessary, multidisciplinary management may further improve overall recovery potential (46). However, the aCCI score also has limitations. Use of the aCCI score alone may underestimate risk in younger patients with multisystem comorbidities, and the assigned weights may not fully reflect actual risk across different disease spectra (47). Thus, in clinical practice, aCCI should be interpreted in conjunction with age, baseline functional status and other risk markers. Overall, aCCI functions not only as a quantitative measure of comorbidity burden but also as an important integrative indicator of expected benefit from reperfusion therapy and rehabilitation potential in AIS. Future work should aim to combine dynamic health scores such as aCCI with multisystem physiological parameters to develop comprehensive risk assessment models that can better inform individualized treatment strategies in AIS.

### 4.5 Atrial fibrillation

AF is characterized by rapid, disorganized atrial electrical activity that leads to ineffective atrial contraction, blood stasis and thrombus formation within the atria. Embolization of these thrombi can result in large-vessel occlusive stroke. According to episode duration and spontaneous termination, AF is classified as paroxysmal, persistent or permanent, and both paroxysmal and persistent AF have been shown to be strongly associated with an increased risk of thromboembolic events (48). In this study, concomitant paroxysmal or persistent AF was associated with a markedly increased risk of poor functional recovery after reperfusion (OR = 3.34, 95% CI 1.66–6.73,*p* < 0.001), indicating that AF is an important independent predictor of attenuated treatment benefit. Atrial fibrillation, a major source of cardioembolic stroke, is associated with stroke severity and recurrence, thereby influencing recovery. Kobeissi et al. reported that patients with stroke complicated by paroxysmal or persistent atrial fibrillation had a higher early mortality rate after thrombectomy than those without atrial fibrillation (49). Jiao et al. further found that atrial fibrillation was associated with lower reperfusion success rates and a significantly increased risk of hemorrhagic transformation. Even when recanalization was achieved on imaging, functional outcomes remained significantly worse in patients with atrial fibrillation than in those without (50). Consistent with previous studies, our findings further support the view that atrial fibrillation may adversely affect functional recovery after reperfusion through multiple mechanisms, including impaired cerebral perfusion and reduced neural recovery capacity. However, the prognostic impact of atrial fibrillation may be heterogeneous. Some studies have suggested that, in certain younger patients, paroxysmal atrial fibrillation may represent a transient reactive state with relatively limited effects on hemodynamic stability and reperfusion efficacy, and thus a weaker influence on long-term functional recovery. This finding indicates that more refined risk assessment based on atrial fibrillation type, duration, and burden may be needed in clinical practice (51). Overall, AF is not only an independent risk factor for poor functional recovery after AIS reperfusion therapy but also a key mechanistic determinant of reperfusion quality and long-term neuroplasticity. Future studies should integrate brain–heart co-imaging and continuous rhythm monitoring to develop dynamic prediction models and to optimize individualized treatment strategies for patients with AF-related stroke.

### 4.6 Common factors without significant effects after reperfusion therapy in acute ischemic stroke: analysis and rationale for retention or exclusion

In this study, traditional risk factors such as age, hypertension, smoking, and alcohol consumption did not show an independent significant association with functional improvement after reperfusion therapy for acute ischemic stroke (*p* > 0.05), although these factors have been widely linked to stroke occurrence and overall prognosis in previous studies. This finding may be related to the specific characteristics of the study population, which consisted exclusively of patients undergoing reperfusion therapy, as well as to the sample composition, stratification features, standardized stroke center workflows, and individualized perioperative management. Because outcomes after recanalization are more directly influenced by proximal factors, including baseline neurological deficit severity, infarct core burden, metabolic status, comorbidity burden, and reperfusion-related factors, the effects of some traditional risk factors may have been attenuated or absorbed in the multivariable model. Further investigation is needed to clarify these relationships in light of sample structure, potential collinearity among variables, and treatment-related mediating pathways.

In addition, the lack of statistical significance for some variables in the multivariable model, despite their selection in the LASSO procedure, may reflect shared variance with other predictors, potential interactions, or sample variability. Nevertheless, these variables were initially retained during the variable selection process because of their established clinical relevance in stroke epidemiology and their potential contribution to overall model stability.

#### 4.6.1 Possible explanations for the reduced impact of age on functional recovery after recanalization therapy in AIS

Further Patients of different ages may differ in physiological reserve, vascular elasticity, inflammatory response, and neuroplasticity. Older patients are also more likely to have multiple comorbidities, poorer collateral circulation, and reduced rehabilitation capacity. Accordingly, age has generally been regarded as an important factor influencing prognosis after reperfusion therapy (52). However, in the present study, age was not independently associated with functional recovery after recanalization (*p* > 0.05). This may be related to cohort characteristics, variable overlap, and improvements in contemporary stroke care. Cho et al. suggested that sample selection and age distribution may weaken the detectable effect of age (53). Patients eligible for reperfusion therapy are typically selected using strict criteria, including treatment time window, imaging findings, and baseline functional status. As a result, extremely elderly or high-risk patients may be underrepresented. In addition, the effect of age may be partly mediated through variables more directly related to outcome, such as admission NIHSS score, baseline ASPECTS score, infarct burden, reperfusion quality, and complications. The inclusion of the aCCI score may also further attenuate the independent effect of age.

Moreover, advances in stroke center management may have reduced the adverse effect of age on short-term outcomes. Therefore, age alone may not fully determine functional improvement after reperfusion therapy. Greater emphasis should be placed on variables more directly associated with prognosis, such as admission NIHSS score, baseline ASPECTS score, complications, and overall health status.

#### 4.6.2 Possible explanations for the reduced impact of chronic diseases on functional recovery after recanalization therapy in AIS

This study found that traditional chronic conditions, including hypertension, as well as a history of diabetes and dyslipidemia, were not independently associated with functional improvement after recanalization therapy in the multivariable model (*p* > 0.05). This does not negate their established roles in stroke occurrence and long-term vascular risk, but suggests that, in the setting of post-recanalization recovery, their effects may be mediated through more proximal acute-phase factors and partly attenuated by standardized contemporary management. For example, the impact of hypertension may be more directly reflected by admission NIHSS score, baseline ASPECTS score, reperfusion status, and hemorrhagic transformation risk (54). Once these variables are included in the model, the independent effect of chronic disease history may be reduced. In addition, standardized perioperative management of blood pressure and glucose may further lessen the short-term influence of these conditions on functional outcome (55). Therefore, chronic disease history may be better regarded as background risk information, whereas acute-phase manifestations, such as blood pressure and glucose fluctuations, inflammatory and coagulation activity, no-reflow, hemorrhagic transformation, and organ dysfunction, may be more directly related to outcome after recanalization.

#### 4.6.3 Possible explanations for the reduced impact of lifestyle factors on functional recovery after recanalization therapy in AIS

Smoking and alcohol consumption are major modifiable risk factors for stroke. Previous studies have largely implicated them in vascular endothelial injury, increased thrombogenicity, accelerated atherosclerosis progression, and poor prognosis (56, 57). However, in this study, smoking and drinking histories did not show independent significance in the multivariable model (*p* > 0.05). This may be attributed to the specific nature of the recanalization treatment cohort, confounding factors, and the measurement methods of variables. First, the impact of smoking or drinking on stroke outcomes often exhibits pronounced confounding structures. Smokers may be younger, have fewer comorbidities, and possess better baseline function, leading to unstable effect directions when adjustments are inadequate. After incorporating key variables such as NIHSS scores, baseline ASPECTS scores, and aCCI scores, their independent contributions may be offset (58). Second, smoking and drinking variables are typically recorded as binary (yes/no), failing to capture true exposure intensity. Factors like daily cigarette consumption, smoking duration, alcohol intake volume and type, and cessation duration remain unmeasured, obscuring dose–response relationships and weakening statistical associations (59). Third, in reperfusion therapy scenarios, short-term functional improvement is directly determined by factors such as ischemic core burden, penumbra salvageability, reperfusion timing, microcirculatory perfusion, and complications. Smoking and alcohol consumption primarily influence these proximal processes indirectly through long-term vascular damage. Once proximal variables are included in the model, the independent predictive effect of lifestyle factors often becomes weaker. Furthermore, smoking and alcohol consumption may be subject to selection bias. Clinically, reperfusion is more readily performed in patients meeting imaging and time-window criteria with better baseline function. This process may exclude or reduce the proportion of high-exposure, high-risk individuals before they enter the reperfusion cohort. While intervention against smoking and alcohol consumption remains crucial at the stroke onset level, their independent effects in predicting short-term functional improvement after reperfusion may be superseded by more direct disease course phenotypes and treatment process variables.

In summary, several traditional factors, including age, hypertension, smoking, and alcohol consumption, did not show independent significance in the multivariable analysis of this study. This may be related to the selection characteristics of the recanalization cohort, overlap and mediation among variables, and improvements in perioperative management in modern stroke centers. These findings suggest that predictive models of benefit after recanalization should place greater emphasis on key phenotypes reflecting the acute disease course and tissue reversibility, such as NIHSS score, ASPECTS score, metabolic status, comorbidity burden, and treatment-related complications. Future studies should further clarify the roles of traditional factors under different outcome definitions and treatment settings by using more refined exposure measures, including blood pressure and glucose control status, smoking and alcohol exposure, dynamic monitoring indicators, and external validation.

## 5 Limitations analysis and future prospects

Several limitations should be considered when interpreting the findings of this study. First, this was a single-center retrospective study with a relatively moderate sample size, which may introduce selection bias and information bias. Although internal validation was performed using a random 7:3 split, the validation cohort was derived from the same single-center dataset. Therefore, this should be regarded as internal rather than external validation. The present results support internal reproducibility only and should not be interpreted as evidence of broad generalizability or clinical readiness.

Patients who died before enrollment or during follow-up were excluded because paired mRS data required for ΔmRS calculation were not available. We acknowledge that death corresponds to an mRS score of 6 and represents the worst functional outcome. Excluding these patients may have underestimated poor functional recovery and introduced survivor bias. Because reliable follow-up information for these patients was unavailable in this retrospective dataset, death could not be assigned as 3-month mRS = 6 in the main analysis or in a sensitivity analysis. This issue should be addressed in future prospective studies.

Second, as an observational study, residual or unmeasured confounding cannot be completely excluded despite adjustment for multiple covariates. In addition, some potentially relevant variables, particularly quantitative imaging biomarkers such as infarct core volume and perfusion parameters, were not included due to the retrospective nature of the study and lack of standardized imaging protocols, which may have affected the predictive performance of the model.

Third, although ΔmRS was used as a dynamic indicator of functional improvement, it is not yet a universally established outcome measure and may be influenced by inter-rater variability and differences in rehabilitation adherence. Moreover, it reflects functional change rather than structural brain recovery, and the lack of standardized follow-up imaging limited further analysis of lesion evolution and its relationship with functional outcomes.

Finally, no formal sample size calculation or statistical power analysis was performed prior to the study due to its retrospective design. Although the current sample size may be adequate for exploratory model development, the absence of *a priori* sample size estimation may affect the statistical robustness of the findings. Future large-scale, multicenter, and prospective studies are warranted to further validate and refine the model.

The cohort included patients treated with intravenous thrombolysis alone, mechanical thrombectomy alone, and bridging therapy. These treatment approaches may differ in baseline severity, workflow, reperfusion success, and recovery pattern. Because the number of patients and outcome events in each treatment subgroup was limited, subgroup-specific model validation was not performed. Combining different reperfusion modalities may therefore limit the interpretation of the model in specific treatment populations.

In the future, from a clinical perspective, the proposed prediction model may serve as a practical tool for early risk stratification in patients with acute ischemic stroke undergoing reperfusion therapy. All variables included in the model are routinely available at admission, allowing clinicians to rapidly estimate the risk of poor functional recovery at an early stage of treatment. In real-world clinical settings, the nomogram can be applied by neurologists or interventional physicians to input individual patient data and obtain an individualized risk probability. Patients identified as high-risk may benefit from closer monitoring, optimization of perioperative management, and early initiation of rehabilitation strategies, whereas patients with lower predicted risk may follow standard treatment pathways. In addition, this model may facilitate clinician–patient communication by providing quantitative prognostic information and assist in clinical decision-making and resource allocation. However, further external validation and integration into clinical decision-support systems are required before widespread implementation.

## 6 Conclusion

Based on routinely available admission clinical, laboratory, and imaging data, this study developed an admission-based pre-reperfusion model to predict poor functional recovery at 3 months after reperfusion therapy in AIS patients. Results indicate that admission NIHSS score, baseline ASPECTS score, admission blood glucose level, aCCI index, and concomitant paroxysmal or persistent atrial fibrillation are independent predictors of poor functional recovery after reperfusion therapy. The model showed acceptable discrimination and calibration in the internal validation cohort and may help with preliminary risk stratification. ΔmRS was calculated as admission mRS minus 3-month mRS and was used as a directional indicator of functional improvement. Compared with relying solely on traditional endpoints such as the 90-day mRS, ΔmRS may help characterize functional change before and after treatment in a more refined manner and provide supplementary information for individualized recovery assessment. Although its clinical applicability and optimal cutoff value still require further validation in larger and external cohorts, this indicator offers an exploratory dynamic perspective for prognostic evaluation after reperfusion therapy in acute ischemic stroke. However, because this was a single-center retrospective study with internal validation only, and because death outcomes and treatment-modality-specific validation were not fully addressed, external validation is required before broader clinical use.

## Data Availability

The raw data supporting the conclusions of this article are available from the corresponding author upon reasonable request. Researchers who require access to the original data should contact the corresponding author directly.
